# Supplementary material for: Knock-out of Tpm4.2/Actin Filaments Alters Neuronal Signaling, Neurite Outgrowth, and Behavioral Phenotypes in Mice
Source: Mol Neurobiol. 2025 Aug 2;62(12):16316–41. doi: 10.1007/s12035-025-05259-9 (PMC12559059; doi:10.1007/s12035-025-05259-9)
Supplement: Supplementary file 1 — Supplementary file1 (DOCX 3.64 MB) [file 12035_2025_5259_MOESM1_ESM.docx]

**Supplementary ~~Information~~ Table and Figures**


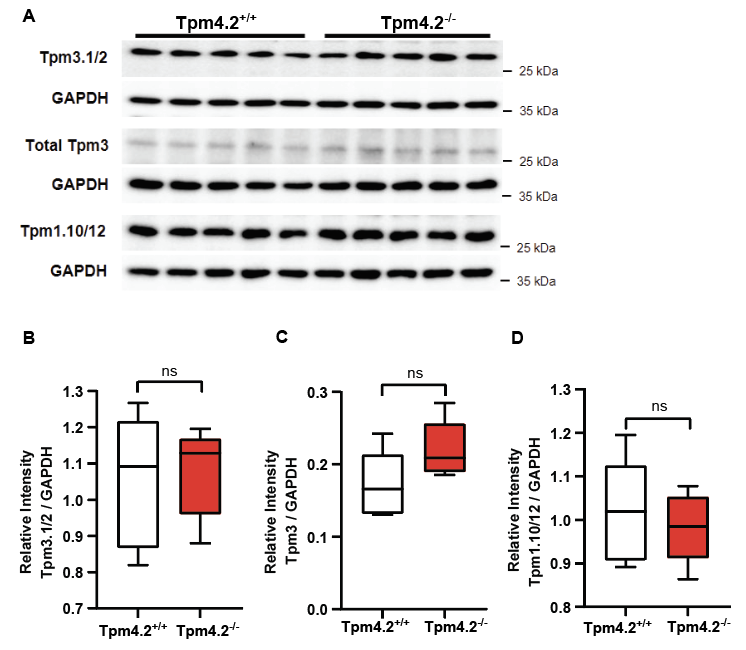


***Figure S1 Assessing compensation of other Tpm isoforms in Tpm4.2^-/-^ mouse brains A*** *Immunoblots, probing for Tpm3.1/2, total Tpm3 isoforms and Tpm1.10/11 in both Tpm4.2^+/+^ and Tpm4.2^-/-^ mouse brains.* ***B-D*** *Data are represented as min-max box plots, n = 5 per group, and statistical analysis, using unpaired t-tests. There was no significant difference in the relative abundance of* ***B*** *Tpm3.1/2,* ***C*** *total Tpm3 isoforms or* ***D*** *Tpm1.10/12 in Tpm4.2^-/-^, compared with Tpm4.2^+/+^ brains (p > 0.05). ns = not significant.*

**Table S1.** Relative abundance of Tpm4.2 in brain regions of Tpm4.2^+/+^ mice.


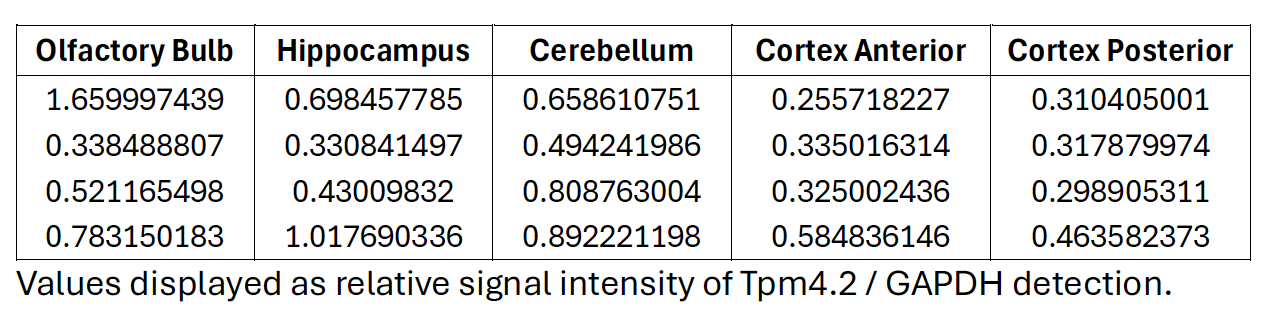


*
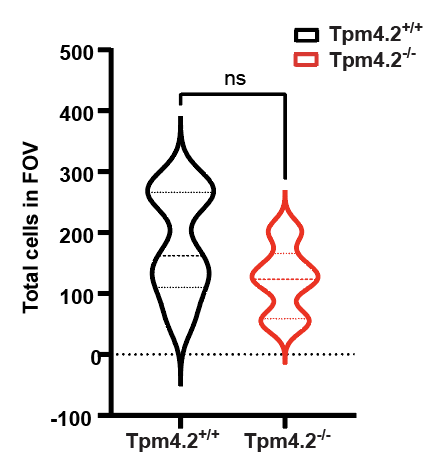
*

***Figure S2 Cell count of cells per field of view (FOV), that were analyzed by calcium imaging.*** *There was no significant difference in the cell density of Tpm4.2^+/+^ and Tpm4.2^-/-^ neurons in the fields of view that were analyzed for calcium spikes.*


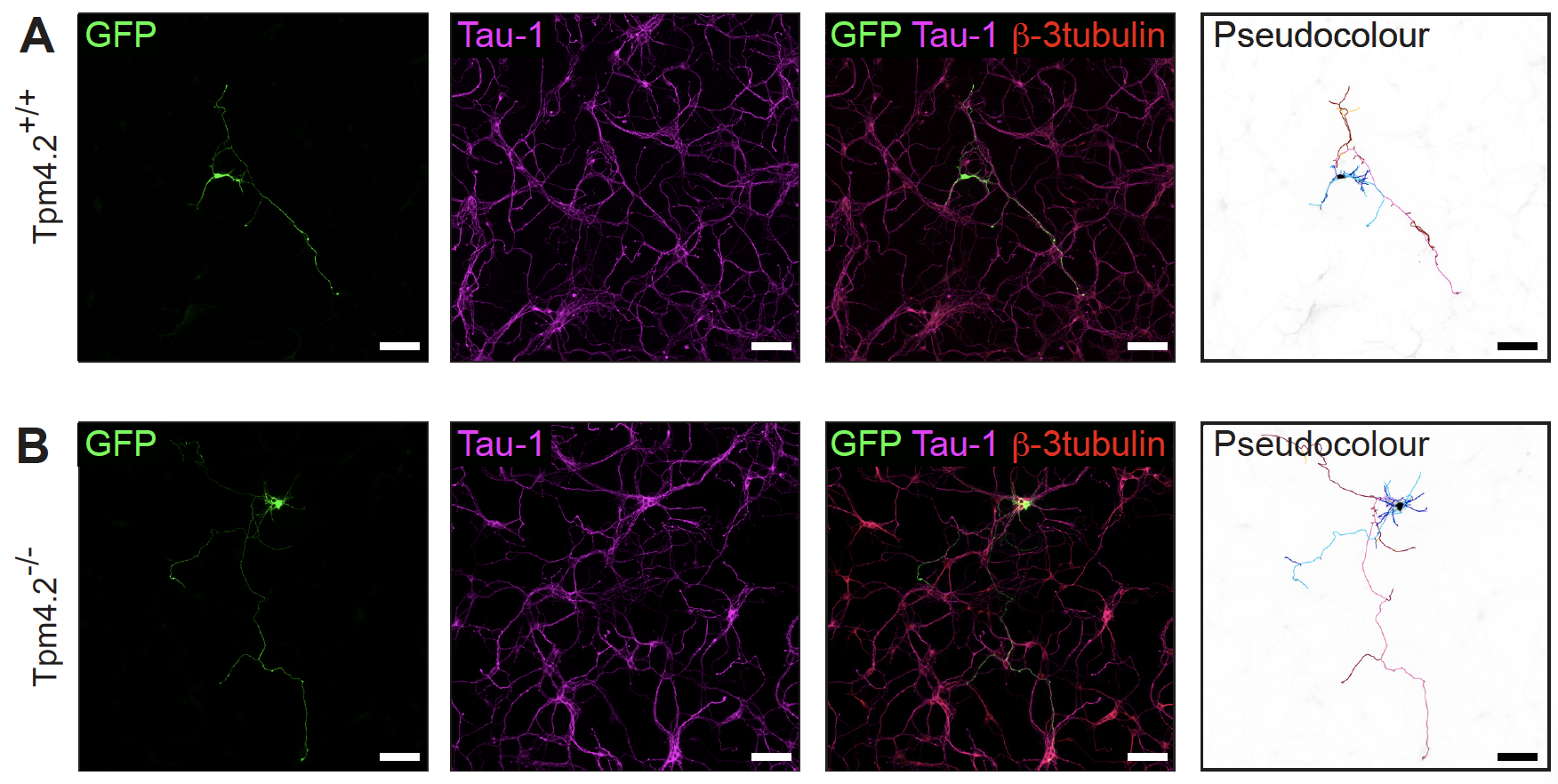


***Figure S3 effects of Tpm4.2 KO on axonal morphology of primary mouse hippocampal***

***neurons.*** *Representative fluorescent images of Tpm4.2^+/+^ and Tpm4.2^-/-^ mouse primary hippocampal*

*neurons, expressing pEGFP-C1. Pseudo-images show the outline of neurites in represented neurons. Curves*

*in pink – axonal shaft, burgundy – primary axonal branches, orange – secondary axonal branches, light blue*

*– dendritic shaft, dark blue – primary dendritic branches, purple – secondary dendritic branches.*

***
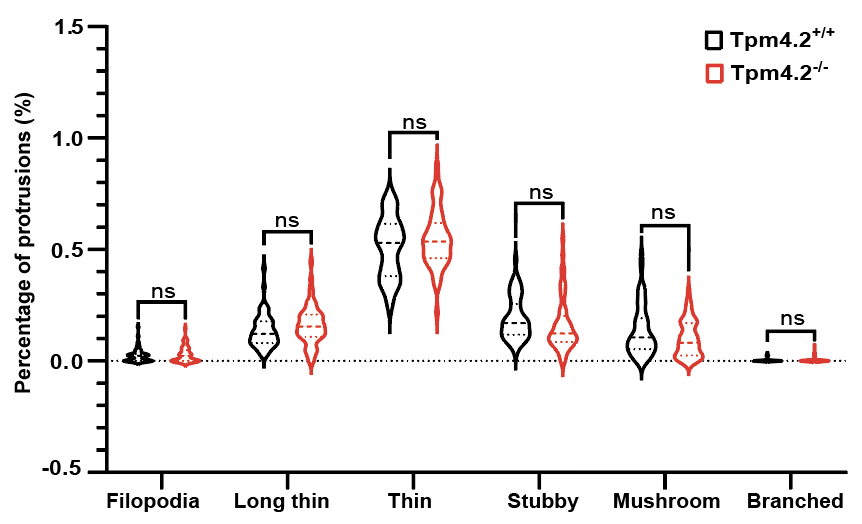
***

***Figure S4 Spine categories for Tpm4.2^-/-^ neurons*** *There were no significant differences in spine categories between Tpm4.2^+/+^ and Tpm4.2^-/-^ neurons. There was a trend towards a decrease in stubby dendritic spines in Tpm4.2^-/-^ neurons p = 0.06. ns = not significant.*


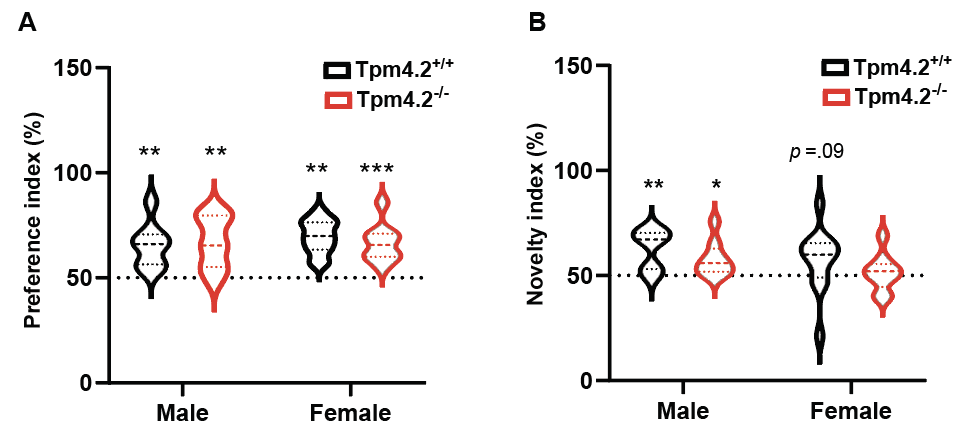


***Figure S5 Sociability test behaviours –*** ***Social preference and social novelty*** *Percentage of time spent nosing enclosure, containing* ***A*** *an A/J mouse versus no mouse (i.e. empty) (sociability) or* ***B*** *a novel versus a familial A/J mouse (social novelty preference), represented by the exploration index (social preference test: mouse x 100/ mouse + empty or social novelty test: new mouse x 100/ new + familiar). Data are shown as mean ± SEM for male and female Tpm4.2^+/+^ (black) n = 25 mice, Tpm4.2^-/-^ (red) n = 20 mice.*


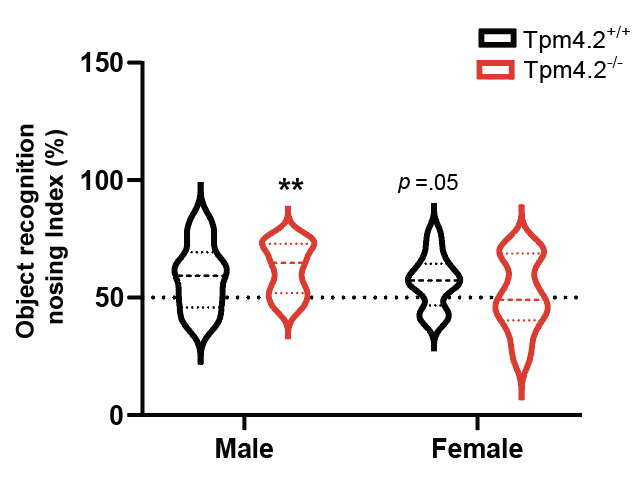


***Figure S6*** ***Novel object recognition behaviours - recognition memory*** *Nosing index, represented by the time exploring the new object as a percentage of total object exploration (%). Data are shown as mean ± SEM for male and female wild type-like Tpm4.2^+/+^ and Tpm4.2^-/-^ littermates.*

**
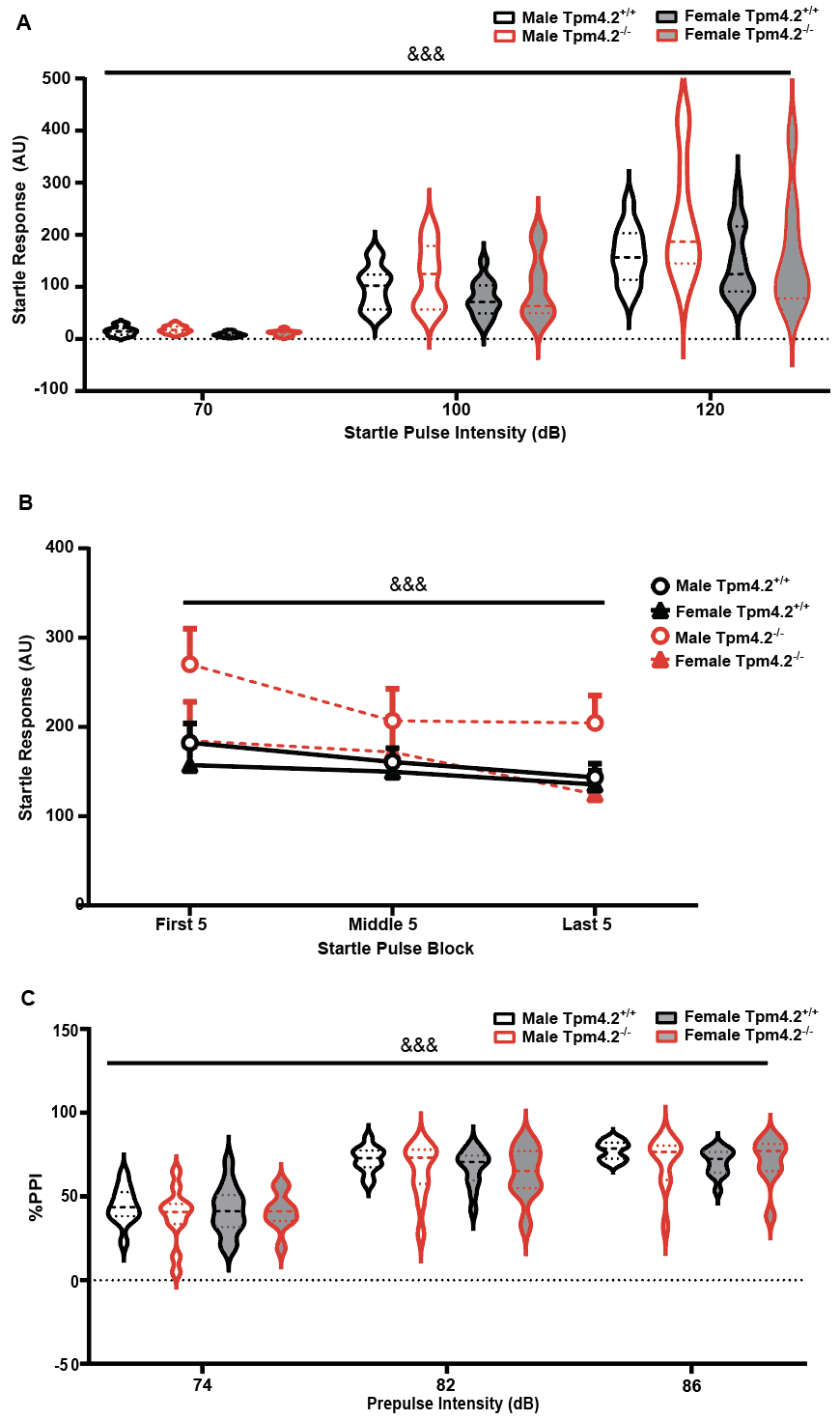
**

***Figure S7 Prepulse Inhibition test in Tpm4.2^-/-^ mice A*** *Average startle response to different pulse intensities.* ***B*** *Startle habituation separated by 5-minute blocks.* ***c)*** *Average percentage of inhibition by different prepulse intensities. Data are shown as mean ± SEM for male and female Tpm4.2^+/+^ (black) n = 25 mice, Tpm4.2^-/-^ (red) n = 20 mice littermates.* Three-way repeated measures ANOVA yielded a significant main effect of ‘startle intensity’ (A), ‘time’ (B) and ‘prepulse intensity’ (C), *&&& p < 0.001*


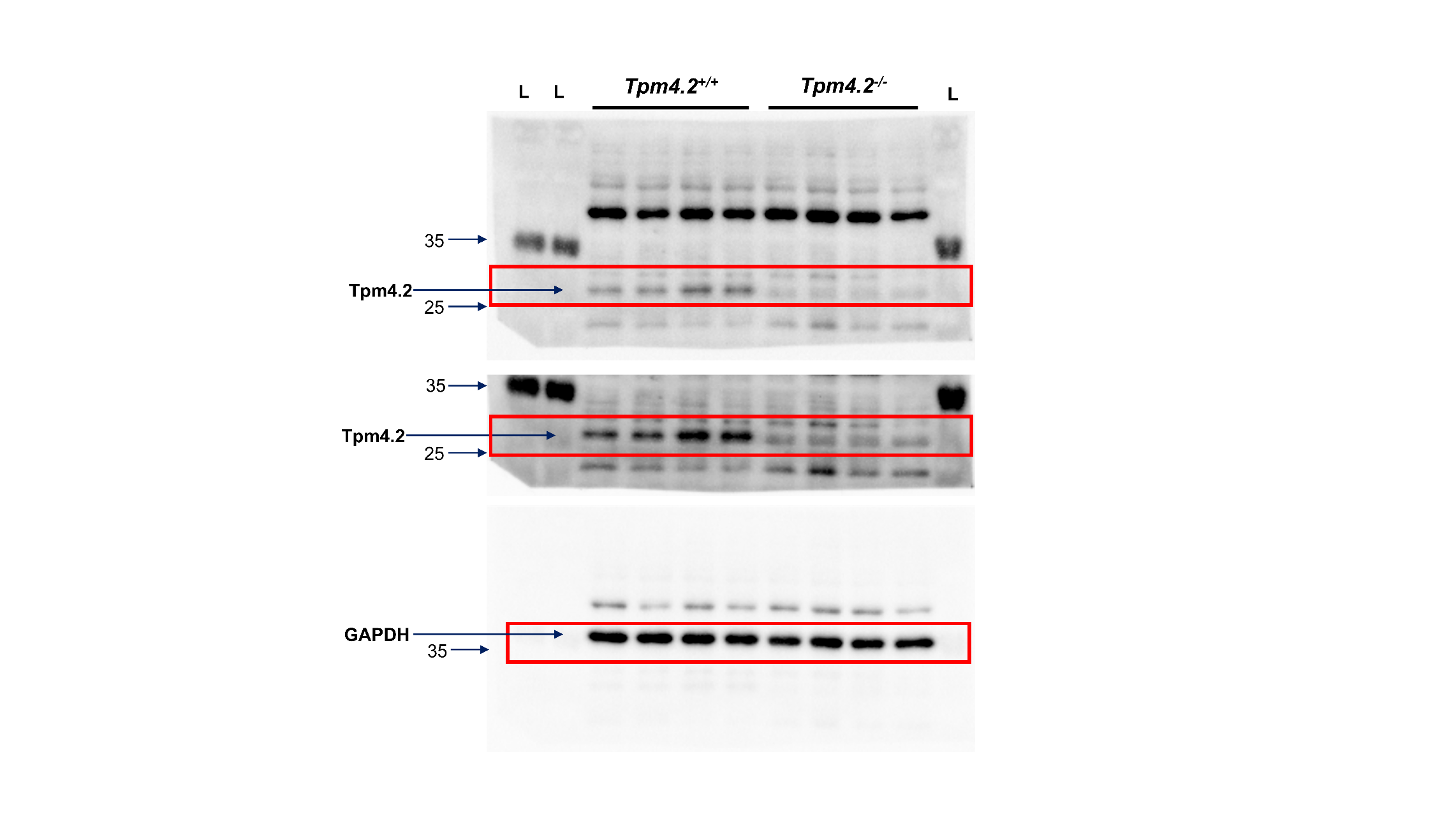


***Figure S8. Validation of total knock-out of Tpm4.2 in Tpm4.2^-/-^ mouse brains.*** *Complete immunoblot results of brains from Tpm4.2^+/+^ and Tpm4.2^-/-^ mice probed for Tpm4.2 (28kDa) and normalized to Glyceraldehyde 3-phosphate dehydrogenase* *(GAPDH; 36kDa). [First lane shows the complete blot; second lane displays the same blot from 35kDa and below with higher exposure to enhance signal intensity of the Tpm4.2-specific bands. The third lane shows the GAPDH of the respective blot].*


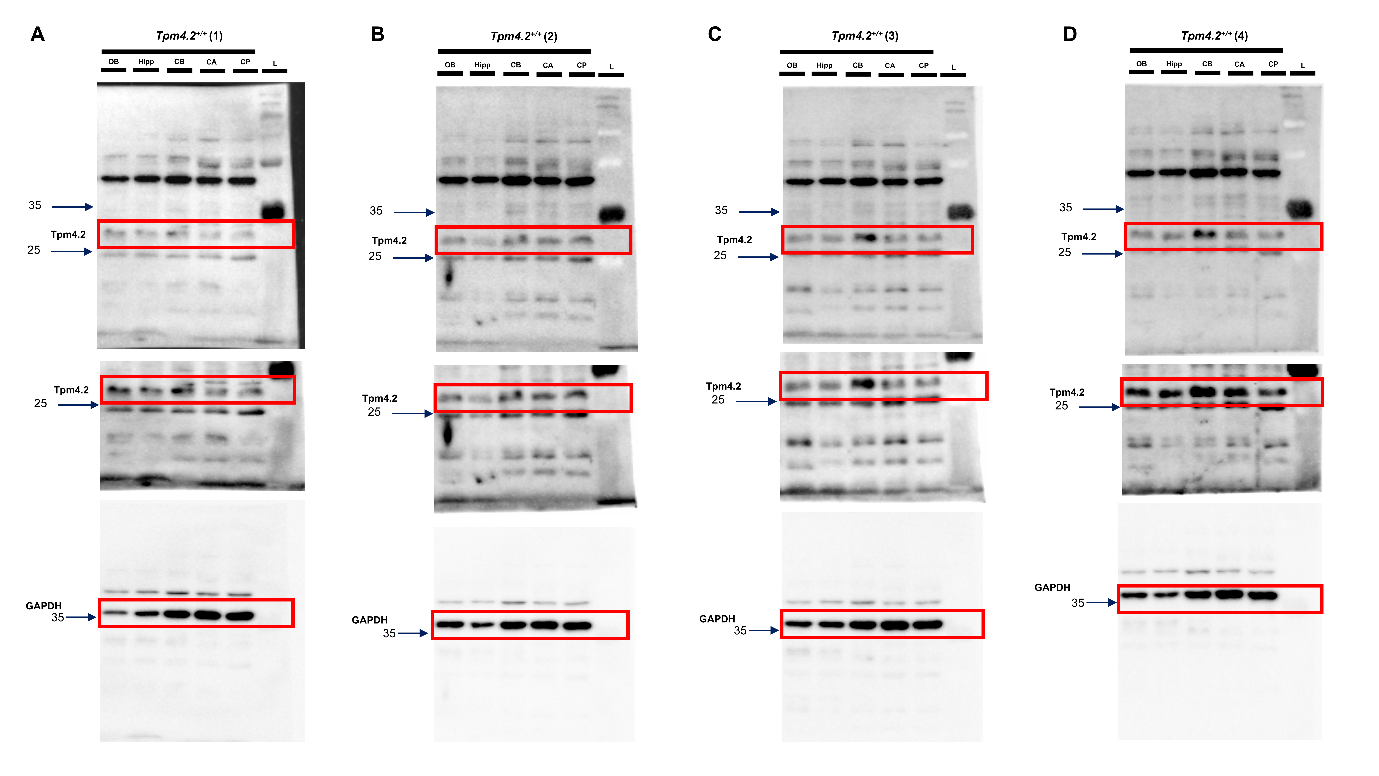


***Figure S9. Complete images of the blots for Figure 1, Tpm4.2 expression in the Tpm4.2^+/+^ mouse brain.*** *Immunoblot results of sub-dissected brains from Tpm4.2^+/+^ mice probed for Tpm4.2 (28kDa) and normalized to GAPDH (36kDa). Olfactory bulb (OB), Hippocampus (Hipp), Cerebellum (CB), Anterior cortex (CA), Posterior* *cortex (CP).* ***A-D.*** *Blots of the different brain regions of four different mice. [First row consists of the complete blots; second row comprises the same blots from 35kDa and below with higher exposure to enhance signal intensity of the Tpm4.2-specific bands. The third row shows the GAPDH of the respective blots].*


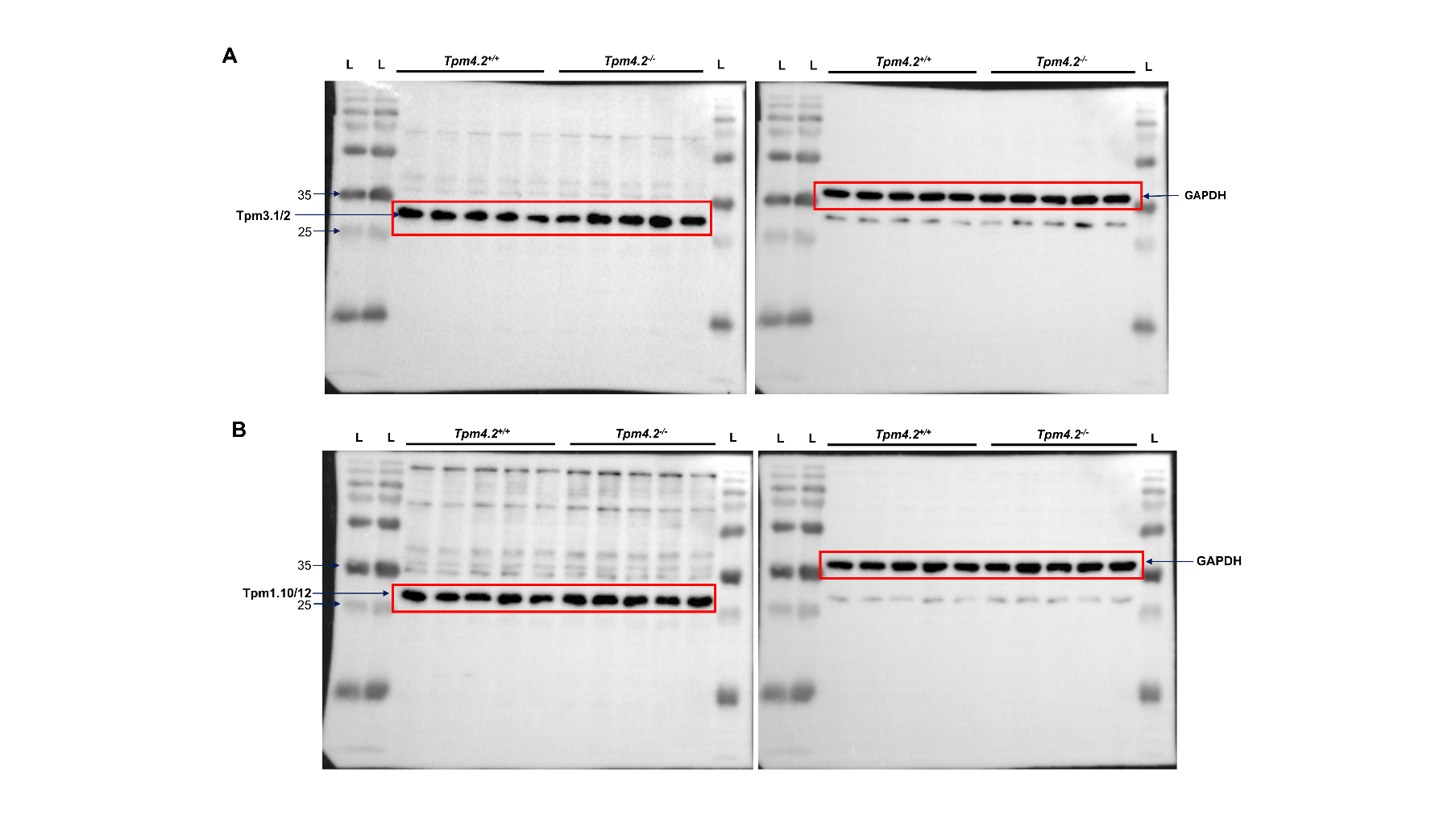


***Figure S10. Complete images of the blots for Figure S1, Assessing compensation of other Tpm isoforms in Tpm4.2^-/-^ mouse brains.*** *Immunoblots results of probing for* ***(A)*** *Tpm3.1/2 and* ***(B)*** *Tpm1.10/11 in both Tpm4.2^+/+^ and Tpm4.2^-/-^ mouse brains. [First column consists of the complete blots; and the second column shows their respective GAPDH].*
